# Endostar in combination with postoperative adjuvant chemotherapy prolongs the disease free survival of stage IIIA NSCLC patients with high VEGF expression

**DOI:** 10.18632/oncotarget.19114

**Published:** 2017-07-08

**Authors:** Zhiwei Chen, Qingquan Luo, Zhen Zhou, Hong Jian, Shun Lu, Meilin Liao

**Affiliations:** ^1^ Shanghai Lung Tumor Clinical Medical Center, Shanghai Chest Hospital, Shanghai Jiao Tong University School of Medicine, Shanghai, China

**Keywords:** cisplatin, non-small cell lung cancer, endostar, VEGF

## Abstract

**Purpose:**

The aim of this study is to compare the therapeutic effect between endostar plus adjuvant chemotherapy and adjuvant chemotherapy alone in the patients with completely resected non-small cell lung cancer (NSCLC) at stage IB to IIIA.

**Experimental Design:**

This is an open, multicenter, randomized (1:1) study with 250 NSCLC patients. Completely resected NSCLC patients at stages IB to IIIA were randomized to receive adjuvant NP plus endostar (Vinorelbine 25 mg/m^2^ on day 1 and day 8 plus Cisplatin 75 mg/m^2^ on day 1, and plus endostar 7.5 mg/m^2^ per day iv for consecutive 14 days) or NP regimen alone. Every 21 days were set as one cycle for 4 cycles. The primary endpoint was disease-free survival (DFS). Secondary endpoints included tumor response rate, overall survival and safety.

**Results:**

The two groups had no significant difference in the incidence of toxicity reaction. Endostar plus NP prolonged the DFS of patients with completely resected NSCLC at stage IIIA (19.33±3.73 vs 17.10±9.68 months) but with no statistical difference compared to NP alone. In the endostar plus NP group, those cases with high expression of vascular endothelial growth factor (VEGF) showed a significantly better DFS than those with low VEGF expression (48.45±3.52 vs 40.18±4.54 months, P<0.05).

**Conclusions:**

Vascular targeted therapy with endostar plus NP prolongs the DFS of patients with complete resectable NSCLC in stage IIIA and significantly extends the DFS of NSCLC patients with high VEGF expression, but does not show benefits in OS for stage IB−IIIA.

## INTRODUCTION

Lung cancer is the leading cause of cancer-related death worldwide [1]. Non-small cell lung cancer (NSCLC) accounts for approximately 85% of lung cancers and its five-year survival rate is below 20% [2, 3]. Chemotherapy is one of the standard therapeutic approaches for advanced NSCLC. Cisplatin-based treatment is the first line chemotherapy for lung cancer. Although advances in cisplatin-based chemotherapy have resulted in improvement of survival rate, the therapeutic efficacy is limited due to the development of cisplatin resistance. For NSCLC patients suitable for tumor resection, postoperative adjuvant chemotherapy could extend the time to recurrence and increase the survival rate of NSCLC patients [4, 5]. However, drug-resistance and over-treatment phenomena are present in most patients receiving conventional postoperative adjuvant chemotherapy [6-8], it is thus important to develop novel postoperative adjuvant chemotherapies to improve the survival rate of operable NSCLC patients.

Angiogenesis plays important roles in various normal physiological processes and deregulation of angiogenesis has been found in several pathological conditions and many human diseases [9, 10]. Angiogenesis is a complicated process that is regulated by many angiogenic factors [11]. Vascular endothelial growth factor (VEGF) and basic fibroblast growth factor (FGF2) are the best-studied angiogenic factors and participate in lots of biological programs, including embryonic development, tumorigenesis, and angiogenesis [12, 13]. Sustained angiogenesis is hallmark of cancer and targeting angiogenesis is a common strategy for development of cancer treatments [14]. Endostatin is a 20-kDa C-terminal fragment derived from type XVIII collagen and is a natural anti-antiogenic molecule. Endostatin is an inhibitor of VEGF and FGF2, and have being widely used for treatment of various cancers [15-19]. Endostar is a derivative of human endostatin modified with 9 amino acids at the N-terminus. The modification increases the stability, prolongs the half-life, and still maintains the biological activity of endostatin. Endostar was approved by SFDA in 2005 and has been used as the first-line therapy for advanced NSCLC combined with chemotherapy in China. Preclinical data revealed that Endostar could inhibit tumor angiogenesis and growth [20]. In a phase III trial, patients with advanced NSCLC were treated with cisplatin/vinorelbine (NP) plus endostar or placebo, the addition of endostar to NP regimen resulted in higher response rate, clinical benefit rate and longer median time to progression compared with NP alone [21, 22]. However, the effects of the adjuvant NP regimen with or without endostar in early-stage NSCLC remain to be determined.

In this study, we enrolled 250 completely resected NSCLC patients at stages IB to IIIA and compared the curative effect of endostar plus adjuvant chemotherapy and adjuvant chemotherapy alone.

## MATERIALS AND METHODS

### Ethical statement

This study was reviewed and approved by the Ethics Committee of Shanghai Lung Tumor Clinical Medical Center, Shanghai Chest Hospital, Shanghai Jiao Tong University, School of medicine. Informed consent was obtained from each patient.

### Patients

In this study, 250 patients, who were diagnosed with NSCLC and treated with surgery between July 2007 and Jun 2009 at Shanghai Chest Hospital, China, were recruited. The inclusion criteria were: histologically confirmed NSCLC; pathologic stage IB-IIIA with complete resection; aged 18 to 70 years, with physical condition score ECOG of 0-1; receipt of chemotherapy 8 weeks after surgery; without signs of tumor recurrence prior to adjuvant chemotherapy. The exclusion criteria were: pathological types did not meet the inclusion criteria; with a history of second malignancies; receipt of preoperative neoadjuvant chemotherapy; pregnancy, or breast-feeding. The NSCLC patients were staged according to the 6th Edition of lung cancer staging developed by American Joint Committee on Cancer (AJCC) and the International Union Against Cancer (UICC) in 2002. The histological diagnosis of each patient was according to the lung and pleura tumor histological type standard set by World Health Organization (WHO) in 2004. Complete resection operation was according to 2007 Non-small cell lung cancer clinical practice guidelines (Chinese Version). The clinical characteristics of the patients in the study are shown in Table 1.

**Table 1:** Clinical characteristics of the patients in the study

Characteristics		NP (*n* = 125)	NP plus Endostar (*n* = 125)	*P*
Age — yr				
Stage I *n* = 118	Range	36∼71	40∼71	0.871
	Mean±SD	56.46±9.00	56.20±7.90	
	Median	58	55	
Stage II *n* = 50	Range	42∼73	37∼70	0.03
	Mean±SD	59.08±8.04	53.84±8.51	
	Median	58	56	
Stage III *n* = 82	Range	33∼75	40∼71	0.19
	Mean±SD	58.41±9.12	55.98±7.50	
	Median	58.5	55.5	
Sex — no. (%)				
Stage I	Male	32 (54.2%)	40 (67.8%)	0.186
	Female	27(45.8%)	19 (32.2%)	
Stage II	Male	20 (80.0%)	18 (72.0%)	0.742
	Female	5 (20.0%)	7(28.0%)	
Stage III	Male	31 (75.6%)	26 (63.4%)	0.337
	Female	10 (24.40%)	15 (36.6%)	

Pathology								
I	Adenocarcinoma		44	(74.6%)		36	(61.0%)	0.30
	SCC		10	(16.9%)		14	(23.7%)	
	Other		5	(8.5%)		9	(15.3%)	
II	Adenocarcinoma		7	(28.0%)		15	(60.0%)	0.064
	SCC		16	(64.0%)		8	(32.0%)	
	Other		2	(8.0%)		2	(8.0%)	
III	Adenocarcinoma		20	(44.8%)		30	(73.2%)	0.077
	SCC		14	(34.1%)		8	(19.5%)	
	Other		7	(17.1%)		3	(7.3%)	
Lung lobe resection								
		One lobe	98		(78.4%)	102	(81.6%)	0.636
		Two lobes	27		(21.6%)	23	(18.4%)	
Treatment Cycles								
1			11				8.80%	8
2			6				4.80%	12
3			12				9.60%	9
4			96				76.80%	96

SCC: Squamous Cell Carcinoma. The number of cycles of adjuvant chemotherapy in both groups was similar (chi-square test, *P* = 0.411). In stages I and III the mean age of the two groups was similar (*t* test, *p* > 0.05); in stage II, the age of the NP group was significantly higher than that of the NP plus Endostar group (59.08 ± 8.04 vs 53.84 ± 8.51, *t* test, *P* = 0.03). The gender ratio was similar between the two groups (chi-square test, *P* <0.05). In both groups, 76.8% of the patients received 4 cycles of adjuvant chemotherapy.

### Therapy programs

The patients were randomly divided into two arms: chemotherapy regimen alone (NP) and adjuvant NP plus Endostar (NP+ENDU). NP program was Vinorelbine (25 mg/m^2^ on day 1 and day 8) plus Cisplatin (75 mg/m^2^ on day 1). NP+ENDU program was Vinorelbine (25 mg/m^2^ on day 1 and day 8) plus Cisplatin (75 mg/m^2^ on day 1), and plus endostar (7.5 mg/m^2^ per day i.v. for consecutive 14 days). Every 21 days were set as one cycle for 4 cycles (Table 1).

### Follow-up

Each patient was scheduled for follow-up visits every two weeks and subjected to chest CT and abdominal B ultrasound, and physical examination to determine the occurrence of relapse. The primary endpoint was disease-free survival (DFS). Secondary endpoints included tumor response rate, overall survival and safety.

### Immunohistochemical analysis

After removal from the human body, all tumor tissue samples were fixed in 4% paraformaldehyde for 24 h and embedded in paraffin. Immunohistochemical analysis was performed on 4 μm-thick sections. Paraffin-fixed tissue sections were deparaffinized twice with xylol for 15 min, and rehydrated with graded alcohol. After blocking endogenous peroxidase with 3% hydrogen peroxide for 10 min, the slides were subjected to antigen retrieval for 5 min in a pressure cooker using sodium citrate buffer (pH 6.0), containing 0.1 M citric acid and 0.1 M sodium citrate in distilled water. After cooling to room temperature, sections were washed twice in PBS. Non-specific binding was blocked by incubating the sections with normal goat serum. Then the slides were incubated with the rabbit polyclonal antibodies against human VEGF (Cell Signaling Technology, Boston, MA, USA) at 1:50 dilution in PBS at 4 °C overnight. The next day after washing with PBS, the sections were incubated with secondary HRP conjugated goat anti-rabbit antibody (Cell Signaling Technology) and hydrogen peroxide for 30 min. Following repeated washing with PBS, the sections were visualized using the ABC substrate buffer for 2 min. Tissue sections were counterstained with hematoxylin, and dehydrated in an ascending series of ethanol (85-100%). After xylol treatment, sections were mounted. As a control, duplicate sections were stained without primary antibodies. Positive cells showed a brownish color. The number and staining intensity of the positive cells were observed by the image analyzer. VEGF staining was assessed by the number of positive tumor cells and staining intensity. Negative or tissues with less than 20% positive cells were defined as VEGF negative; while strong staining with more than 20% positive cells were defined as VEGF positive.

### Statistical analysis

The primary end point was disease-free survival after randomization. Secondary end points were overall survival and adverse effects. The events considered in disease-free survival were locoregional or distant recurrences and death without a recurrence. Median follow-up was estimated with the use of the Log-rank tests. All analyses were performed strictly according to the intention-to-treat principle and included all randomized patients, eligible or not. For the main analysis of overall survival, we used a Cox model adjusted according to previously defined stratification factors (center, stage of disease, and type of surgery). For secondary analyses, we used Cox models to study variations in treatment effects according to major base-line characteristics (age, sex, performance status, type of surgery, stage of disease, pathological nodal stage, and histologic findings) and treatment options. The incidence rates of safety events were compared with the use of Fisher’s exact test. All reported P values are two-sided. P-values<0.05 were considered statistically significant. Data were analyzed with the use of SPSS software, version 21.0.

## RESULTS

### Treatment cycles and VEGF expression status

The two arms were well balanced with regard to age, gender, histology, staging, and resection type. Both arms were planned for 4 cycles of 21 days in each cycle. In each group of the 125 patients, 96 patients finished 4 cycles of treatment. The other 29 patients in each group received 1, 2 or 3 cycles of treatment due to different reasons (Table 1). VEGF expression status as determined by immunohistochemical analysis is shown in Supplementary Table 1.

### Disease free survival

**Figure 1:**
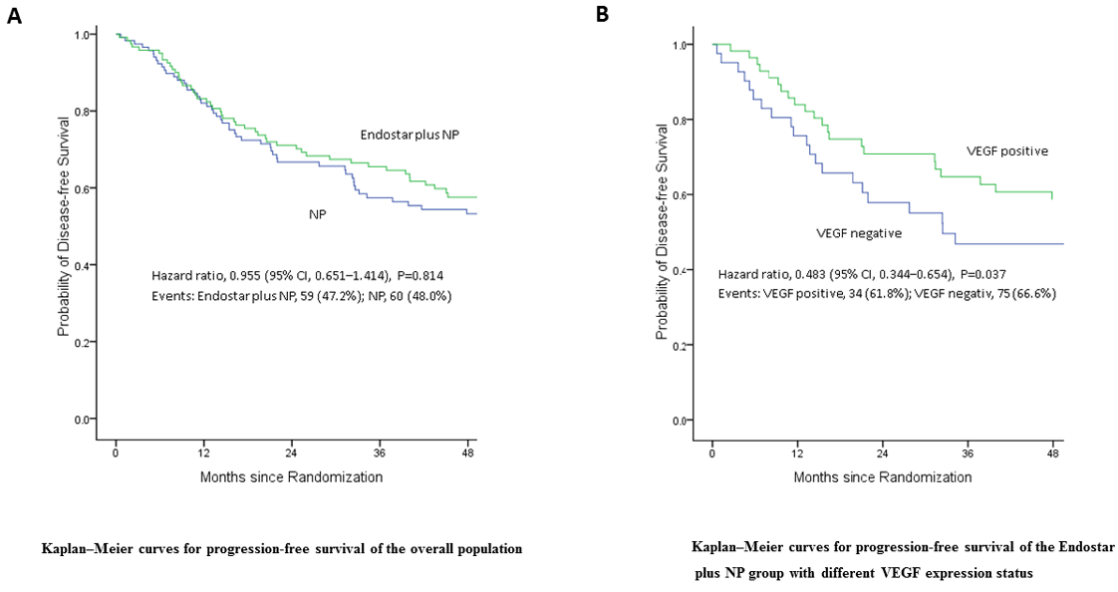
Disease free survival (DFS) of patients following treatment **A.** Endostar plus NP did not significantly increase the DFS of patients with complete resectable NSCLC (*p* = 0.814). **B.** Endostar plus NP significantly increases the DFS of patients with high expression of VEGF (*p* = 0.037).

The DFS of patients in NP arm with complete resectable NSCLC at stage IIIA was 17.10±9.68 months, while the DFS of Endostar plus NP was 19.33±3.73 months, indication of prolonged DFS (Table 2 and Figure 1A). However there was no statistical difference (*P* = 0.6). Therefore, endostar plus NP did not significantly increase the DFS of patients with complete resectable NSCLC.

**Table 2:** The disease free survival data

	NP plus Endostar (*n*=125)		NP (*n*=125)			Hazard ratio (95% CI)	Log Rank p
	*n*	DFS (Median )	*n*		DFS (Median)		
Stage I	59	>60m		59	>60m	0.788 (0.419-1.478)	0.457
Stage II	25	>60m		25	>60m	0.695 (0.279-1.729)	0.431
Stage III	41	20.4m		41	17.1m	1.153 (0.687-1.936)	0.590

As of 60 months of follow-up, the two groups did not reach Median DFS in stages I and II; Phase III reached the median DFS (hazard ratio for disease progression or death in the NP plus Endostar group was 1.153; 95 percent confidence interval, 0.687 to 1.936; Log Rank test, *P* > 0.05). There was no signficant difference in DFS between the two treatments.

We then sub-grouped the patients in Endostar plus NP arm to VEGF positive and negative groups according to the VEGF expression status and analyzed DFS. The results demonstrated that the DFS of VEGF positive patients was 48.45±3.52 months, while the DFS of VEGF negative patients was 40.18±4.54 months, with the p value of 0.037 (Figure 1B and Table 3). Thus, endostatin plus NP significantly increased the DFS of patients with high expression of VEGF.

**Table 3:** The DFS and OS of patients with VEGF expression status

	NP plus Endostar		NP	
	*n*	DFS (Median)	*n*	DFS (Median)
VEGF(+)	55	48.45m	65	46.36m
VEGF(-)	70	40.18m	60	45.04m
Hazard ratio (95% CI)		0.483(0.344–0.654)		0.629 (0.358-1.100)
Log Rank P		0.037		0.104

	NP plus Endostar			NP	
	(*n* =125)			(*n* =125)	
		5 Yr(%)	OS (Median)	5 Yr(%)	OS (Median)
VEGF(+)		66	>60m	53	>60m
VEGF(-)		66	>60m	66	>60m
Hazard ratio (95% CI)		1.068(0.543–2.099)		0.618(0.336-1.135)	
Log Rank P		0.849		0.121	

Up to 60 months of follow-up, the median DFS in VEGF-positive expression was signifiantly higher than that in the negative expression of VEGF in the NP plus Endostar group (48.45m vs 40.18m; hazard ratio for disease progression or death in the NP plus Endostar group was 0.483; 95 percent confidence interval, 0.344 to 0.654); The expression of VEGF in NP group was not correlated with DFS (hazard ratio 0.629 (0.358-1.100) , Log Rank test , *P* > 0.05). The median OS in VEGF-positive expression was similar to that in VEGF negative in the NP plus Endostar group (hazard ratio 1.068 (0.543–2.099) , Log Rank test , *P* > 0.05); The expression of VEGF in NP group was not correlated with OS (hazard ratio 0.618 (0.336-1.135, *P* > 0.05). The OS of the two groups did not reach the median.

### Overall survival

**Figure 2:**
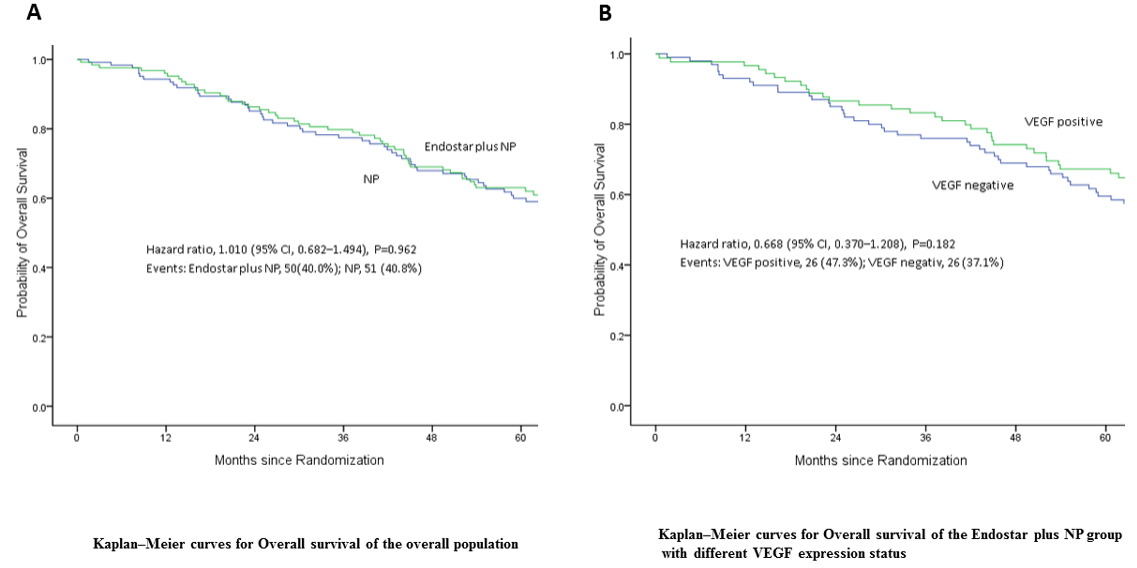
Overall survival (OS) of patients following treatment **A.** Endostar plus NP prolonged the OS of patients with complete resectable NSCLC but with no statistical significance (*P* = 0.962). **B.** Endostar plus NP did not significantly increase NSCLC patient’s OS regardless expression status of VEGF (*P* = 0.182).

The follow-up time was 60 months. The OS of patients with complete resectable NSCLC at stage IIIA in NP arm was 39.53±9.23 months, while that in Endostatin plus NP was 41.27±4.24 months, indicating that Endostatin plus NP prolonged OS of average of 1.74 months (Table 4 and Figure 2A). However there was no statistical difference (*P* = 0.76). Therefore, endostatin plus NP did not significantly increase the OS of patients with complete resectable NSCLC.

**Table 4:** The overall survival data

Stage	NP plus Endostar (*n* = 125)		NP (*n* = 125)		Hazard ratio (95% CI)	P (Log Rank)
	*n*	OS (Median )	*n*	OS (Median )		
I	59	>60m	59	>60m	0.952(0.466-1.946)	0.894
II	25	>60m	25	>60m	0.797(0.320-1.982)	0.625
III	41	41.27m	41	39.53m	1.090(0.627-1.893)	0.760

Up to 60 months of follow-up, stages I and II did not reach Median OS, and stage III reached Median OS. The Median DFS in the NP plus Endostar and NP groups was 41.27 months and 39.53 months, repectively. There was no significant difference in the Overall OS between the two groups (Log Rank test, *P* > 0.05).

The patients in both arms were sub-grouped to VEGF positive and negative groups according to the VEGF expression status. Then the 5-year survival rates were analyzed. The 5-year survival rates of NP arm patients were 66% in both VEGF positive and negative patients. The 5-year survival rates of VEGF positive and negative patients in Endostatin plus NP arm were 66% and 53%, respectively, with Log-rank p value of 0.21 (Figure 2B and Table 3). Thus, in endostatin plus NP did not significantly increase NSCLC patient’s OS regardless the expression status of VEGF.

### Adverse reaction

All 250 patients completed at least one cycle of therapy. The most common side effects were neutropenia, anemia and vomitting. There were no stage III or stage IV side effects. The toxicities are summarized in Table 5. The two groups had no significant difference in the incidence of toxicity reaction.

**Table 5:** Treatment-related adverse events

	NP plus Endostar (%)	NP (%)	*P* value
Withdrawal due to any AE	0	0	1.0000
AEs occurring in ≥10% of patients			
Neutropenia	82	74	0.8813
grade 3/4	38	31	0.7570
Anemia	54	42	0.5813
grade 3/4	6	3	0.6171
Thrombocytopenia	31	26	0.8364
grade 3/4	2	2	0.8807
Vomiting	67	74	0.8948
grade 3/4	18	23	0.7695
Constipation	17	22	0.7578
grade 3/4	1	1	0.7772
Cardiac disorders				
arrhythmia	5	8	0.7196
thromboembolic event	1	0	1.0000
hypertension	1	1	0.7772
phlebitis	1	0	1.0000

The Common Terminology Criteria (CTC) grade is defined on the basis of the National Cancer Institute Common Terminology Criteria for Adverse Events, version 4.0. The incidence of AE was similar in both groups, and there was no AE-resulted in withdrawal. The incidence of AE in cardiac disorders was <10%, and the levels were grade 1/2.

## DISCUSSION

Angiogenesis is essential for malignant tumors to grow and metastasize. VEGF plays a pivotal role in neovascularization during tumorigenesis [9, 10]. Under normal conditions, VEGF is marginally expressed in a lot of normal tissues, while highly expressed in the tumors like osteosarcoma, bladder cancer, breast cancer and colorectal cancer. Accordingly, anti-angiogenesis based therapy has become one of the major strategies for the treatment of a lot of solid tumors, with several inhibitors targeting angiogenesis especially VEGF/VEGFR signaling pathways in clinical application and numerous agents in pre-clinical development and clinic trials [23, 24]. It has been demonstrated that the combination of chemotherapy plus anti-angiogenesis based therapy such as bevacizumab, a recombinant humanized monoclonal antibody against vascular endothelial growth factor A (VEGF-A), increased the response rate and progression-free survival of patients with NSCLC [25, 26]. The potential mechanism of anti-angiogenic agents combined with adjuvant chemotherapy may include re-establishing the balance of anti-angiogenesis, blocking tumor angiogenesis, inhibiting or delaying residual tumor recurrence [17]. Anti-angiogenic agents combined with adjuvant chemotherapy may also stop the growth in “dormant” micrometastases. Moreover, anti-angiogenic therapy may sensitize tumor cells to chemotherapy, improving the efficacy of adjuvant chemotherapy. In addition, antiangiogenic therapy combined with chemotherapy will likely further delay tumor recurrence and metastasis after surgery time, reduce the rate of tumor recurrence and prolong survival of patients. Endostatin is a naturally occurring, 20-kDa C-terminal fragment derived from type XVIII collagen. Similar to angiostatin and thrombospondin, it has been shown to significantly inhibit tumor proliferation and metastasis [15]. Extensive clinical trials have shown that endostatin can be beneficial in combinations with other medicines, but endostatin alone give no significant improvements in tumor/disease progression [26, 27]. Endostar, a novel recombinant human endostatin, was approved by SFDA in 2005 and has been used as the first-line therapy for advanced NSCLC combined with chemotherapy in China. Increasing preclinical and clinical data have shown that endostar could sensitize advanced NSCLC to cisplatin/vinorelbine (NP) [20-22]. However, whether endostar plus adjuvant chemotherapy may improve the survival of early stage NSCLC patients after surgery is unknown.

In this study, we enrolled 250 patients with completely resected NSCLC patients at stages IB to IIIA and randomized them to receive adjuvant NP plus Endostar or NP regimen alone. The patients were followed up for 5 years. Endostar plus NP prolonged the DFS of patients with complete resectable NSCLC at stage IIIA to average of 2.23 months with high safety, however there was no statistical difference between the two arms, suggesting that endostar plus NP does not significantly increase the DFS of patients with complete resectable NSCLC. In addition, though Endostar plus NP prolonged OS of average of 1.74 months but with no statistical difference, indicating that endostar plus NP does not significantly increase the OS of patients with complete resectable NSCLC. Very intriguingly, our results demonstrated that Endostar plus NP prolonged the DFS of VEGF positive patients with complete resectable NSCLC at stage IIIA to average of 8.27 months with statistical significance when compared to VEGF negative patients (*p* = 0.037). Our findings showed that endostar plus NP significantly increased the DFS of patients with high expression of VEGF.

In summary, vascular-targeted therapy with endostar could prolong the DFS of patients with complete resectable NSCLC in stage IIIA and significantly extended the DFS of NSCLC patients with high expression of VEGF, but did not show benefits in OS for stage IB−IIIA. With the advance of precision medicine, endostar plus NP may be used for the treatment of NSCLC patients with high expression of VEGF.

## SUPPLEMENTARY MATERIALS TABLE


