# Supplementary material for: Endostar in combination with postoperative adjuvant chemotherapy prolongs the disease free survival of stage IIIA NSCLC patients with high VEGF expression
Source: Oncotarget. 2017 Jul 8;8(45):79703–11. doi: 10.18632/oncotarget.19114 (PMC5668083; doi:10.18632/oncotarget.19114)
Supplement: Supplementary file 1 [file oncotarget-08-79703-s001.pdf]

Endostar in combination with postoperative adjuvant chemotherapy prolongs the disease free survival of stage IIIA NSCLC patients with high VEGF expression

SUPPLEMENTARY MATERIALS

Supplementary Table 1: VEGF expressions status as analyzed by immunohistochemistry (IHC)

Groups		VEGF (+)	VEGF (-)	P
I	NP	53%	47%	1.00
	NP + ENDU	53%	47%	
II	NP	37%	63%	0.53
	NP + ENDU	50%	50%	
III	NP	37%	63%	0.29
	NP + ENDU	54%	46%	

There was no significant difference in the positive expression rate of VEGF between the two groups (IHC method) (chi-square test, P <0.05). VEGF staining was assessed by the number of positive tumor cells and staining intensity. Negative or tissues with less than 20% positive cells were definced as VEGF (-); while strong staining with more than 20% positive cells were definced as VEGF (+).
